# Stereotactic radiotherapy of intracranial tumor beds on a ring‐mounted Halcyon LINAC

**DOI:** 10.1002/acm2.14281

**Published:** 2024-01-26

**Authors:** Joshua Misa, Shane McCarthy, William St. Clair, Damodar Pokhrel

**Affiliations:** ^1^ Medical Physics Graduate Program Department of Radiation Medicine University of Kentucky Lexington Kentucky USA

**Keywords:** AcurosXB, Brain SRT, Co/non‐planar geometry, FFF‐beam, Halcyon RDS, VMAT

## Abstract

**Purpose:**

This study sought to evaluate the feasibility and efficacy of the Halcyon Ring Delivery System (RDS) for delivering stereotactic radiotherapy (SRT) treatments for intracranial tumors beds.

**Methods:**

Ten previously treated brain SRT patients for 30 Gy in five fractions with non‐coplanar HyperArc plans on TrueBeam (6MV‐FFF) were replanned on Halcyon (6MV‐FFF) using the same number of arcs and Eclipse's AcurosXB dose engine. Plan quality evaluation metrics per SRT protocol included: PTV coverage, GTV dose (minimum and mean), target conformity indices (CI), heterogeneity index (HI), gradient index (GI), maximum dose 2 cm away from the PTV (D_2cm_), and doses to organs‐at‐risk (OAR). Additionally, patient‐specific quality assurance (QA) results and beam‐on‐time (BOT) were analyzed.

**Results:**

The Halcyon RDS provided highly conformal SRT plans for intracranial tumor beds with similar dose to target. When benchmarked against clinically delivered HyperArc plans, target coverage, CI(s) and HI were statistically similar. The Halcyon plans saw no statistical difference in maximum OAR doses to the brainstem, spinal cord, and cochlea. Due to the machine's coplanar geometry, the Halcyon plans showed a decrease in optic pathway dose (0.75 Gy vs. 2.08 Gy, *p* = 0.029). Overall, Halcyon's coplanar geometry resulted in a larger GI (3.33 vs. 2.72, *p* = 0.008) and a larger D_2cm_ (39.59% vs. 29.07%, *p* < 0.001). In this cohort, multiple cases had the PTV and the optic pathway in the same axial plane. In one such instance, the PTV was <2 cm away from the optic pathway but even at this close proximity OAR, Halcyon still adequately spared the optic pathway. Additionally, the Halcyon's geometry provided slightly larger amount of normal brain dose receiving 24.4 Gy (8.99 cc vs. 7.36 cc) and 28.8 Gy (2.9 cc vs. 2.5 cc), although statistically insignificant. The Halcyon plans achieved similar delivery accuracy, quantified by patient‐specific QA results evaluated with a 2%/2 mm gamma criteria (99.42% vs. 99.70%). For both plans, independent Monte Carlo second checks calculation agreed within 1%. Average Halcyon BOT was slightly higher by 0.35 min (*p* = 0.045), however, due to the one‐step patient set‐up and verification overall estimated treatment times on Halcyon were lower compared to HyperArc treatments (7.61 min vs. 10.26 min, *p* < 0.001).

**Conclusions:**

When benchmarked against clinically delivered HyperArc treatments, the Halcyon brain SRT plans provided similar plan quality and delivery accuracy but achieved faster overall treatment times. We have started treating select brain SRT patients on the Halcyon RDS for patients having tumor beds greater than 1 cm in diameter with the closest OAR distance of greater than 2 cm away from the target. We recommend other clinics to consider commissioning SRT treatments on their Halcyon systems–allowing including remote Halcyon‐only clinics to provide exceptionally high‐quality therapeutic brain SRT treatments to an otherwise underserved patient cohort.

## 1 INTRODUCTION

Brain metastases are a very common diagnosis, with the rate of incidence increasing due to technological and medical innovations leading to a longer patient survival than previously thought possible. Typical brain metastase treatments involve surgical resection followed by radiation therapy. Radiation therapy is especially important for large, intracranial metastases to dramatically improve local control with multi‐modality treatments.^1^ Traditionally, adjuvant therapy consisted of whole brain radiation therapy (WBRT), which decreases the incidence of recurrence within the tumor bed. In the past few decades, stereotactic radiotherapy, and radiosurgery (SRT/SRS) have been validated and clinically used to treat such intracranial tumor beds. Studies have shown that SRT is a viable alternative to WBRT,^2^, ^3^ achieving better tumor local control compared with surgery alone while maintaining low rates of radiation‐induced toxicity.^4^, ^5^ However, tumor local control has been shown to be heavily impacted by the size of the tumor bed (resection cavity) volume.^6^, ^7^, ^8^ For these larger targets, a multitude of literature compares hypofractionated SRT (SRT) to single‐shot SRS treatment and revealed that SRT demonstrates excellent local control while demonstrating lower risks of normal brain radionecrosis and other radiation‐induced toxicities.^9^, ^10^, ^11^, ^12^, ^13^, ^14^, ^15^ There has been some use of robotic CyberKnife to deliver brain SRS, opposed to conventional LINAC‐based SRS.^16^ However, CyberKnife SRS report to have higher integral dose compared to conventional LINAC‐based SRS^17^ and CyberKnife units are not readily available to many community cancer centers.

Recently, to provide fast, effective, and high‐quality radiation treatments to underserved communities, Varian Medical Systems (Palo Alto, CA) has introduced the Halcyon V2.0, a new, jawless, single energy, ring delivery system (RDS).^18^ This Halcyon RDS is equipped with a 6MV‐FFF beam with a maximum output of 800MU/min, significantly lower than that of the corresponding C‐arm SBRT‐dedicated 6MV‐FFF TrueBeam LINAC (1400MU/min). The Halcyon's 6MV‐FFF beam yields a lower mean energy of 1.3 MeV and nominal depth of 1.3 cm that could be beneficial for brain treatment compared to that of the TrueBeam's 6MV‐FFF mean energy of 1.4 MeV and nominal depth of 1.5 cm. The lower mean energy contributes to a sharper dose fall‐off, potentially resulting in a reduced dose to organ‐at‐risk (OAR). Due to Halcyon's less rounded MLC tip design (dosimetric leaf gap of 0.1 mm), it results in a reduced beam penumbra. Moreover, its fully interdigitated, faster (maximum up to 5 cm/s), stacked and staggered MLC configuration results in ultra‐low leakage and transmission of 0.45% on the Halcyon RDS. Additionally, the Halcyon includes a one‐step patient set‐up and verification feature, in which the patient is aligned to the virtual blue lasers (at the patient's skin marks) then the isocenter shifts are automatically applied. For image‐guided radiation therapy, Halcyon is equipped with a fast, on‐board imaging capability of a 15‐second iterative kV‐cone‐beam CT (kV‐iCBCT) image reconstruction that provides high‐quality pre‐treatment iCBCT images.^19^, ^20^ Although limited by its three degrees‐of‐freedom (3DoF) couch correction, compared to TrueBeam's 6DoF PerfectPitch couch, it is capable of reproducing isocenter positional accuracy (within ± 0.9 mm)^21^ similar to our TrueBeam LINAC and is well within tolerance for SRT treatments as recommended by TG‐101.^22^

In the use of the SRS/SRT regimen, there have been a limited number of studies performed utilizing the novel Halcyon RDS.^23^, ^24^ For instance, Knutson et al.^23^ investigated the workflow for brain SRT treatments provided by the Halcyon and revealed shorter treatment times with the Halcyon. Another report by Li et al.^24^ performed a retrospective dosimetry planning study investigating Halcyon's capability in treating multiple brain lesions with a single‐isocenter approach. Although these retrospective studies reported similar plan quality of Halcyon for intracranial lesions compared to conventional C‐arm LINACs, they did not treat any patients on Halcyon, yet. As of now, there has not been any published literature that investigates the benchmarking and utilization of the Halcyon in the treatment of intracranial tumor beds, especially the large resection cavities. In this study, we conducted a retrospective benchmarking study of plan quality, delivery accuracy, efficiency, and safety of the Halcyon RDS in delivering brain SRT treatments for intracranial tumor beds following the AAPM TG‐101 recommendations, HyTEC guidelines and brain SRT/SRS Alliance 071801 trial criteria.^10^, ^22^, ^25^ Additionally, we have begun treating intracranial tumor bed patients on our Halcyon LINAC as demonstrated in this technical report as a prospective example brain SRT treatment.

## 2 MATERIALS AND METHODS

### 2.1 Patient characteristics, CT simulation, and target definition

After Institutional Review Board approval from our institute, ten patients with intracranial tumor beds that were treated with HyperArc brain SRT on our TrueBeam LINAC were selected and anonymized for this retrospective study. These patients underwent an SRT regimen of 30 Gy in 5 fractions. For HyperArc brain SRT planning, these patients were immobilized using the Q‐Fix mask, SRS headrest, and mouth‐bite on the Encompass device (Q‐Fix, Avondale, PA) in the headfirst supine position with arms on both sides of the hand grip array. High‐resolution 3D planning CT imaging was obtained on a SOMATOM go.Open Pro CT scanner (Siemens Healthineers, Malvern, PA) with a 512 × 512 pixel image size and 1.0 mm slice thickness using an axial helical scan. This CT scan included central and lateral markers on the Encompass device and included inferiorly to the patient's shoulders to assess any collisional issue. The gross tumor volume (GTV) was derived via gadolinium contrast‐enhanced, high‐resolution magnetization‐prepared rapid gradient‐echo (MP‐RAGE) MRI images that were co‐registered to the planning CT images. The target volume was contoured by an experienced radiation oncologist in which the GTV was delineated by the visible resection tumor bed. The PTV was created with a uniform 2.0 mm margin around the GTV following departmental SRT protocol. The relevant critical structures that were contoured for optimization and dose reporting included: optic pathway, brainstem, spinal cord, cochlea, brain minus PTV, and skin. Tumor bed characteristics and locations are shown in Table 1 for all 10 brain SRT patients used for this retrospective analysis. A variety of tumor bed locations were chosen to further analyze the robustness of SRT treatments on the Halcyon. Patients with a wide range of PTV sizes from 3.8 cc to 94.8 cc, were included in this study to help further analyze the adaptability of Halcyon for the SRT treatments of varying‐sized intracranial tumor beds.

**TABLE 1:** Patient characteristics for brain SRT validation study on the Halcyon RDS: Prescription was 30 Gy in five fractions.

Patient #	GTV (cc)	PTV (cc)	Brain tumor location
1	10.1	19.7	Right frontal
2	2.9	8.2	Left parietal
3	73.8	94.8	Left occipital
4	19.2	48.4	Left frontal
5	27.8	42.0	Left frontal
6	10.8	15.8	Left cerebellum
7	57.1	83.4	Bi‐lateral frontal
8	1.7	3.8	Right cerebellum
9	2.6	5.4	Left temporal
10	15.3	25.6	Bi‐lateral cerebellum
Mean ± SD (range)	22.1 ± 24.5 (1.7−73.8)	34.7 ± 32.3 (3.8−94.8)	

*Note*: These patients were originally treated with HyperArc SRT on a TrueBeam LINAC.

### 2.2 Clinical HyperArc SRT plans

Clinical HyperArc SRT plans for intracranial tumor beds were generated by an experienced SRT physicist using the fully automated non‐coplanar HyperArc Module (on TrueBeam) in Varian's Eclipse TPS (version 15.6). Following SRT recommendations from RTOG protocols, patients were prescribed a dose of 30 Gy in 5 fractions with the D_95%_ of the PTV receiving 30 Gy or higher. All HyperArc SRT plans had their isocenter automatically chosen to be placed in the center of the PTV. This allows for the Virtual Dry Run (in the HyperArc Module) and reduces the risks of patient collision with the fully automated delivery for gantry/couch/collimator rotations on TrueBeam. All HyperArc plans utilized four, non‐coplanar arcs that utilized optimized collimator angles based on the tumor bed's size, shape, and location to minimize MLC leakage dose and improve target conformity. The 6MV‐FFF beam and SRS normal tissue objective (NTO) was used. As per SRT protocol, target conformity and heterogeneity indices, high‐ and low‐dose spillage, and dose‐limiting organ constraints were reported for all patients. The patients were immobilized using Q‐Fix's Encompass SRS Immobilization System with an integraBite mouthpiece as mentioned before. The beam attenuation through the Encompass support device was accounted for in Eclipse TPS. To account for full scatter simulation, the body contour was expanded to include the Q‐Fix equipment, SRS headrest, and mouthpiece.

### 2.3 Halcyon SRT plans

Each patient was retrospectively replanned by an experienced SRT physicist on the Halcyon RDS. All HyperArc plans were then fully optimized for the Halcyon's coplanar geometry with four full 360‐degree arcs. When replanning, the Encompass device was removed from the planning CT structure set and the Halcyon couch structure was inserted. In order to reproduce similar target coverage while maintaining maximum doses to adjacent OAR, the optimization objectives on the Halcyon differed from the clinically HyperArc plans; re‐normalizing Halcyon plans yielded similar PTV coverage as the clinical HyperArc plans. The Halcyon plans were in most cases renormalized to achieve similar PTV V30Gy as the HyperArc plans. Both the clinical HyperArc brain SRT plans and Halcyon plans used Eclipse's AcurosXB dose calculation model with a 1.25 mm dose calculation grid size for tissue heterogeneity corrections selected.^26^, ^27^, ^28^ All physics quality assurance procedures, machine QA checks including machine performance checks, independent isocenter check, and patient‐specific QA results were in compliance with brain SRT treatment delivery as recommended by the AAPM TG‐101.^22^

### 2.4 Planning criteria and plan comparison

The clinical TrueBeam and the Halcyon SRT plans were compared via brain SRT protocol's requirements for PTV coverage, target dose conformity (CI), tumor dose heterogeneity (HI = maximum dose/prescription dose), gradient index (GI), and the Paddick's conformity index were evaluated.^29^ Additionally, the intermediate dose fall‐off was quantified by the maximum dose 2 cm away in any direction from the PTV (D_2cm_). See Table 2 for our departmental brain SRT protocol used in the evaluation of the plan quality between the two SRT delivery platforms. Moreover, all brain SRT plans were evaluated for the volume of normal brain receiving 24.4 Gy (V24.4) and 28.8 Gy (V28.8), respectively. Critical OAR dose metrics were evaluated based on their maximum and volumetric doses as per departmental SRT protocols based on the HyTEC papers, Alliance brain SRS/SRT 071801 trial, and TG‐101 recommendations.^10^, ^22^, ^30^ The dose to OAR evaluated, and protocol metrics are shown in Table 3.

**TABLE 2:** Plan quality metrics for target coverage and intermediate dose fall‐off for 5‐fraction brain SRT applied in our clinic.

Parameter	Definition or volume	Clinical goal (acceptable variation)
PTVD99	Dose received by 99% of PTV	—
Min GTV dose	Minimum dose to GTV	≥ 30 Gy
Mean GTV dose	Mean dose to GTV	—
CI	V30Gy ÷ PTV	1.0−1.2 (1.0−1.5)
Paddick CI	TV^2^_30Gy_ ÷ (TV × V30Gy))	Desirable ∼1.0
HI	Maximum point dose ÷ Prescription dose	1.1−1.4
GI	V15Gy ÷ V30Gy	<3 (<4)
D_2cm_	Maximum dose 2 cm away from the PTV in any direction	≤50% (<60%)

*Note*: Summarized from Alliance trial, HyTEC brain SRT papers, TG‐101 recommendations.

Abbreviations: TV = Treated volume, V30Gy = volume receiving 30 Gy, V15Gy = volume receiving 15 Gy dose.

**TABLE 3:** Summary of plan quality metrics for dose to OAR and the corresponding dose tolerances for 30 Gy in 5‐fraction brain SRT and toxicity concerns reported per Alliance 071801 brain SRT trial, HyTEC brain SRT papers and TG‐101 recommendations.

Critical organs	OAR volume	Clinical goal (acceptable variation)	End point indications (≥ Grade 3 toxicity concern)
Brainstem	D_0.03cc_	<28 Gy (<31 Gy)	Cranial neuropathy
	D_0.5cc_	<23 Gy (<26 Gy)	
Spinal cord	D_0.03cc_	<27 Gy (<30 Gy)	Myelitis
	D_0.35cc_	<23 Gy (<25 Gy)	
Optic pathway	D_0.03cc_	<23 Gy (<25 Gy)	Neuritis
	D_0.2cc_	<20 Gy (<23 Gy)	
Normal brain (Brain‐PTV)	V24.4	<10 cc (<20 cc)	Brain necrosis
	V28.8	<10 cc (<20 cc)	
Cochlea	D_0.03cc_	<25 Gy	Hearing loss

In addition to the aforementioned plan quality metrics, delivery efficiency and accuracy, the total number of monitor units (MU) per fraction, beam modulation factor (MF = ratio of total number of MU per fraction to the prescription dose in cGy), and beam‐on time (BOT) was recorded. Dosimetric verification of both brain SRT plans were performed using a portal dosimetry (PD) quality assurance (QA) procedure established in our clinic. Our clinical gamma criteria for VMAT SRT patient‐specific QA were 2%/2 mm with a low‐dose threshold set to 10% as similar to the previously reported procedures.^25^, ^31^ The electronic portal imaging device (EPID, aS1200 flat panel detectors, Varian Medical Systems, Palo Alto, CA) mounted on the TrueBeam and Halcyon LINACs were used. The EPID detector has an active area of 40 cm × 40 cm with a high‐resolution pixel size of 0.34 mm. Included as part of our clinics SRT QA procedure, an independent physics 2^nd^ check was performed using an in‐house Monte Carlo dose verification. Moreover, overall treatment time was estimated by incorporating the time to set up the patient and perform pre‐treatment CBCT imaging, registration, and applying shifts including dry‐run time and couch rotation times (on TrueBeam) on both platforms. Comparisons of dosimetric parameters and plan delivery metrics between the two brain SRT plans were performed using a paired samples *t*‐test with a statistically significance level of *p*‐value < 0.05.

## 3 RESULTS

### 3.1 Target coverage & intermediate dose spills

For both brain SRT plans, the plan quality metrics for the tumor dose, PTV coverage, and intermediate dose fall‐off is shown in Table 4. All plans were in compliance with TG‐101 requirements, Alliance 071801 brain clinical trial, and HyTEC brain SRT papers as mentioned before. Halcyon plans demonstrated statistically similar GTV doses (minimum and mean), target CI, and the Paddick's CI. As the Halcyon plans had been normalized to achieve similar target coverage as the clinical HyperArc plans, this similarity was expected. However, one of the HyperArc plans had a CI of 0.65 in comparison to the Halcyon replan with a CI of 0.98. In this case, the target was abutting the brainstem. The HyperArc plan prioritized sparing the brainstem (underdosing the target) while the Halcyon replan prioritized target coverage. In this case, the Halcyon plan met the acceptable variation of the maximum dose to brainstem criteria while providing appropriate target coverage. The Halcyon plans resulted in a statistical increase in HI and GI but were still within compliance with brain SRT protocol requirements. The largest statistical difference is revealed when comparing the D_2cm_ metric, in which the Halcyon plans had an increase of 10.52% (*p* < 0.001). The statistical increase in those metrics is most likely attributed to the Halcyon's coplanar geometry compared to the highly non‐coplanar HyperArc geometry.

**TABLE 4:** Evaluation of plan quality metrics for target coverage and intermediate dose fall‐off for 5‐fraction brain SRT treatment on the Halcyon RDS. Mean±SD (range) was reported.

Parameter	HyperArc SRT	Halcyon SRT	*p*‐value
PTVD99 (Gy)	28.71 ± 1.82 (23.72−29.89)	29.64 ± 0.37 (28.85−30.08)	0.098
Min GTV dose (Gy)	30.34 ± 1.24 (28.28−31.88)	30.85 ± 0.75 (29.50−31.81)	0.248
Mean GTV dose (Gy)	33.61 ± 1.56 (31.68−37.31)	33.67 ± 1.20 (32.50−36.57)	0.927
CI	0.97 ± 0.12 (0.65−1.05)	1.03 ± 0.04 (0.98−1.09)	0.141
Paddick CI	0.90 ± 0.09 (0.66−0.96)	0.93 ± 0.02 (0.89−0.96)	0.328
HI	1.18 ± 0.04 (1.11−1.24)	1.22 ± 0.05 (1.15−1.30)	**0.016**
GI	2.72 ± 0.51 (2.12−3.44)	3.33 ± 0.81 (2.56−5.10)	**0.008**
D_2cm_ (%)	29.07 ± 7.02 (15.80−37.70)	39.59 ± 7.18 (28.20−51.00)	**<0.001**

*Note*: Statistically significant p‐values are highlighted in bold.

### 3.2 Dose to adjacent OAR

Maximum and volumetric doses to the brainstem, spinal cord, optic pathways, and cochlea (maximum) were evaluated. Additionally, the volume of the normal brain (brain minus PTV volume) receiving 24.4 Gy and 28.8 Gy was evaluated. The results for both plans are presented in Table 5. The Halcyon SRT plans showed statistically insignificant differences in OAR doses to the brainstem, spinal cord, and normal brain. However, due to the non‐coplanar geometry of HyperArc plans compared to the Halcyon's coplanar geometry, the Halcyon plans achieved a statistically significant decrease in dose to the optic pathways. Although, the absolute differences were very small and maximum dose to optic pathway were within the SRT protocol's recommendations for both platforms. There was an outlier in our study, the previously mentioned case where the target was abutting the brainstem. The Halcyon plan prioritized target coverage dose over the dose to brainstem in the optimizer, resulting in higher maximum doses to the brainstem and the spinal cord but within protocol criteria. For this outlier case, we chose not to renormalize the Halcyon plan to achieve similar coverage as the respective HyperArc plan, as we were able to achieve better target coverage while respecting the dose to OARs. Within this study cohort, we had some cases where the optic pathway was in the same axial plane as the brain tumor bed. One such case had the target less than 2 cm away from the optic pathway. In these cases, the maximum dose to the optic pathways was similar between the two plans. Nevertheless, the Halcyon was still able to demonstrate acceptable maximum dose to the optic pathways while effectively sparing the OAR.

**TABLE 5:** Evaluation of dose achieved to OAR for corresponding dose tolerances for 5‐fraction brain SRT per Alliance trial, HyTEC brain SRT papers, TG‐101 recommendations.

Critical organs	OAR volume	HyperArc SRT	Halcyon SRT	*p*‐value
Brainstem (Gy)	D_0.03cc_	4.95 ± 6.54 (1.37−22.93)	4.95 ± 8.66 (0.14−28.62)	0.997
	D_0.5cc_	4.04 ± 5.71 (1.17−19.98)	4.27 ± 7.73 (0.13−25.62)	0.756
Spinal cord (Gy)	D_0.03cc_	3.28 ± 6.58 (0.08−21.80)	3.04 ± 8.82 (0.04−28.12)	0.760
	D_0.35cc_	2.48 ± 4.52 (0.07−15.11)	2.49 ± 7.25 (0.04−23.11)	0.990
Optic pathway (Gy)	D_0.03cc_	2.08 ± 1.61 (0.29−4.73)	0.75 ± 0.71 (0.09−2.40)	**0.029**
	D_0.2cc_	1.74 ± 1.53 (0.25−4.25)	0.59 ± 0.51 (0.08−1.76)	**0.036**
Normal brain (cc)	V_24.4_ _Gy_	7.36 ± 4.73 (0.62−12.90)	8.99 ± 5.12 (1.94−15.32)	0.065
	V_28.8_ _Gy_	2.5 ± 2.5 (0.00−7.57)	2.9 ± 2.3 (0.39−8.12)	0.202
Cochlea (Gy)	D_0.03cc_	1.80 ± 1.28 (0.66−4.57)	1.39 ± 2.03 (0.07−4.76)	0.438

*Note*: Mean±SD (range) was reported. Statistically significant *p*‐values are highlighted in bold.

### 3.3 Treatment delivery efficiency and accuracy

For both platforms, treatment delivery efficiency was performed for all brain SRT plans by comparing the total MU per fraction, beam MF, BOT, and the estimated overall treatment times. See Table 6 for treatment delivery metrics used and associated values for both the HyperArc and the Halcyon SRT plans.

**TABLE 6:** Evaluation of treatment delivery parameters for HyperArc versus Halcyon plans for brain SRT patients.

Treatment and QA Parameters	HyperArc SRT	Halcyon SRT	*p*‐value
Monitor units per fraction	1978 ± 557 (1324−2892)	2092 ± 380 (1638−2769)	0.515
MLC modulation factor (MF)	3.30 ± 0.93 (2.21−4.82)	3.49 ± 0.63 (2.73−4.61)	0.515
Beam‐on time (min)	2.26 ± 0.35 (2.00−3.06)	2.61 ± 0.48 (2.05‐3.46)	**0.045**
Treatment time (min)	10.26 ± 0.35 (10.00−11.06)	7.61 ± 0.48 (7.05−8.46)	**<0.001**
PD VMAT QA γ pass rate (%)	99.42 ± 0.62 (98.40−100.00)	99.70 ± 0.45 (98.60−100.00)	0.262
MC 2^nd^ physics check pass rate (%)	98.10 ± 1.70 (95.80−100.00)	98.56 ± 1.91 (95.90−100.00)	0.361

*Note*: Statistically significant values are highlighted in bold.

Abbreviation: MC, Monte Carlo.

When comparing treatment delivery efficiency, the Halcyon plans provided similar MU and MF values compared to the clinical HyperArc plans. The Halcyon plans do see a statistically significant increase in BOT by 0.35 min, on average. However, with Halcyon's one‐step patient set up and pre‐treatment verification, the estimated overall door‐to‐door patient times including patient setup and CBCT, provided statistically significant faster (*p* < 0.001) treatment delivery around 7.5 min. Treatment times were estimated based on recorded times of patient setup and kV‐iCBCT imaging and auto‐registration times from previous HyperArc patients and prospective Halcyon patients treated at our institution. The Halcyon compensated for its relatively slower maximum dose rate (800 MU/min), compared to the TrueBeam's (1400 MU/min), with its faster gantry rotation speed of up to 4 revolutions/min and its automated one‐step patient setup and verification. Treatment delivery accuracy was evaluated by patient‐specific QA results as well as an independent dose verification via our in‐house, clinically used MC 2^nd^ check dose calculation algorithm. The Halcyon showed statistically insignificant differences in both patient‐specific PD‐QA and MC 2^nd^ check results, suggesting comparable treatment delivery accuracy.

### 3.4 First brain SRT on Halcyon: Plan quality and treatment deliverability

**FIGURE 1:**
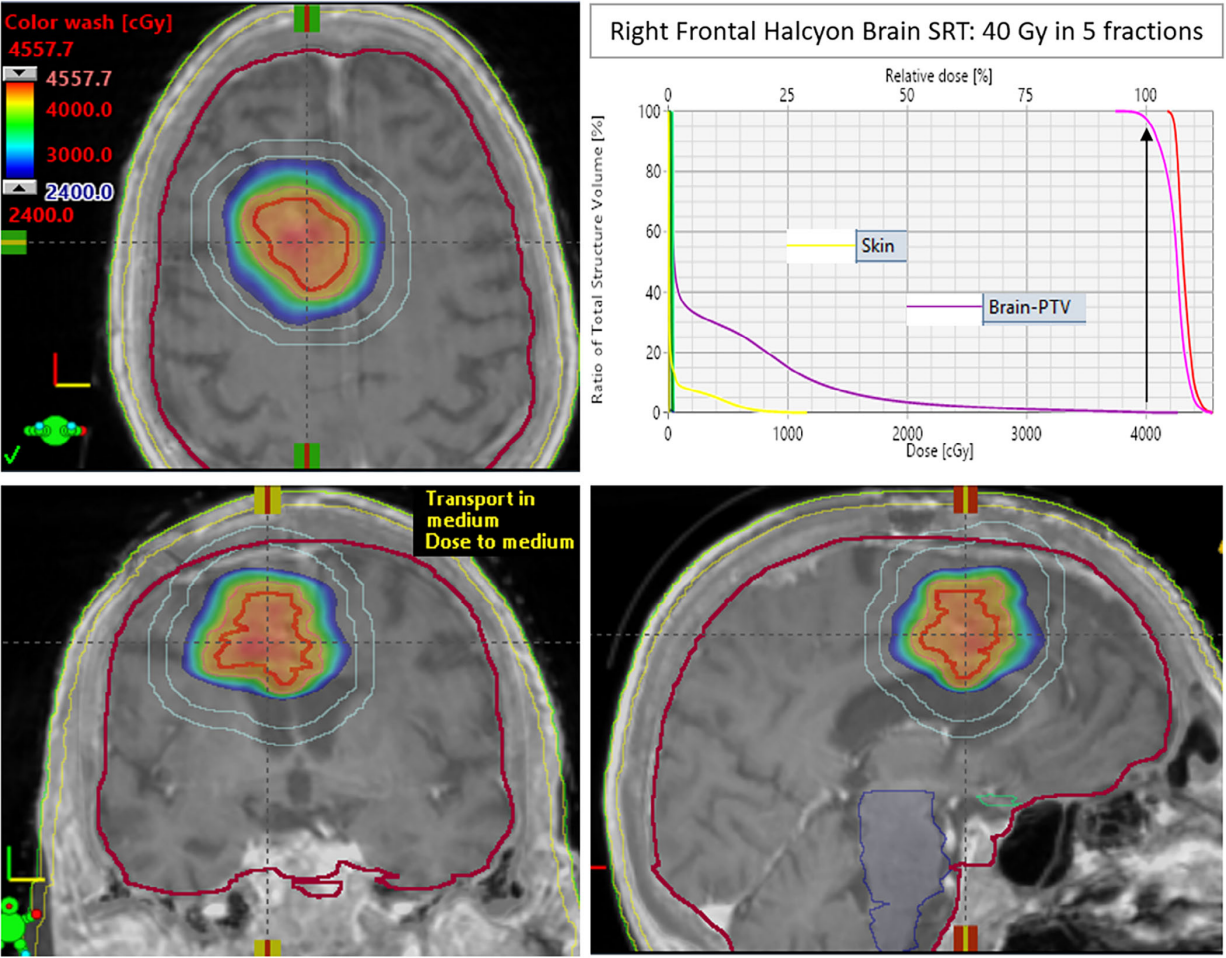
Demonstration of the first clinical example of Halcyon SRT plan (40 Gy in five fractions, vertical black arrow, every other day) for right frontal tumor bed (renal cell carcinoma primary). The GTV (red, 7.5 cc) and PTV (purple, 17.5 cc) received highly conformal dose distribution (CI = 1.02) while sparing normal brain minus PTV (light brown). A tight D_1cm_ ring (sky blue) around the PTV used for dose steering. Lower 60% isodose colorwash is shown for this 40 Gy SRT treatment plan. GI of 3.6 demonstrate that Halcyon's co‐planar geometry provided acceptable intermediate dose fall‐off for this patient.

Based on these promising validation results, we have clinically implemented brain SRT treatment on our Halcyon for select patients who presents with relatively large tumor beds (>1 cm) that are far away (>2 cm or higher) from the critical organs (optic pathway and brainstem). The first patient who underwent brain SRT on the Halcyon was prescribed 40 Gy in 5 fractions treated every other day to a right frontal tumor bed (see Figure 1). This patient had a metastatic brain tumor due to a radiosensitive renal cell carcinoma primary, thus our experienced radiation oncologist decided to prescribe 40 Gy rather than conventional 30 Gy for this tumor bed as benchmarked above. The GTV (red contour, 7.5 cc) was derived from the gadolinium contrast‐ enhanced post‐operative (after 4 weeks) MP‐RAGE MRI images co‐registered with the fine‐resolution (1 mm) planning CT images. The PTV size was 17.4 cc (pink contour, 3.2 cm diameter). Four full coplanar arcs were used each with optimized collimator rotations. The clinical goal was for 95% of the PTV to receive 40 Gy. Our Halcyon plan achieved this with 95% of the PTV receiving 101.1% the prescription dose and a mean GTV dose of 43.2 Gy. Figure 1 shows the delivered brain SRT dose distribution for the right fontal tumor bed in the axial, coronal, and sagittal views through the isocenter plane (crosshair) for our first brain SRT patient treated on the Halcyon RDS.

**FIGURE 2:**
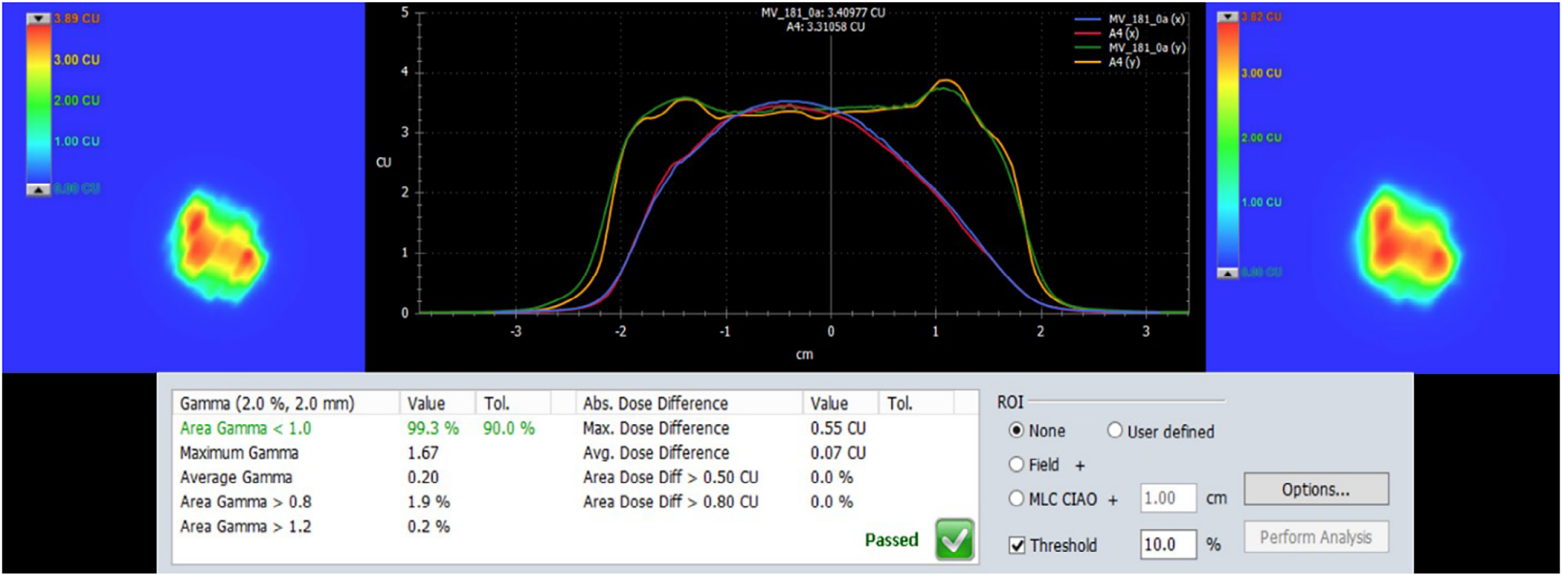
Demonstration of the patient‐specific VMAT PD‐QA performed for the brain SRT patient on the Halcyon. Left panel represents the calculated composite EPID portal fluence map and right panel shows the measured composite portal fluence map. Middle panel shows the vertical dose profile comparison between the two fluences on the Halcyon. Bottom table shows the VMAT PD‐QA pass rate of 99.3% when using a 2%/2 mm clinical gamma criteria.

All DVH parameters were compliant with our clinical SRT protocols. The Halcyon plan achieved a CI of 1.05, HI of 1.14, GI of 3.6, and D_2cm_ of 44.6%. Moreover, the maximum dose to the dose steering ring 1 cm away from the PTV (D_1cm_) was 59.0%. Maximum doses to brainstem, spinal cord, optic pathways, and cochlea were well below clinical tolerances, all <0.5 Gy. As shown in Figure 1, for this given higher prescription dose (40 Gy), the dose to skin and normal brain was also minimized as much as possible. With the lower mean energy of the Halcyon's 6MV‐FFF beam, sharper penumbra, and ultra‐low MLC leakage and transmission, the Halcyon provided a clinically desirable, steep intermediate dose fall‐off with a tight 60% isodose distribution (see blue isodose colorwash in Figure 1) required for brain SRT treatment. Total MU per fraction and MF was 2570 and 3.21, respectively. Before the first fraction of the brain SRT treatment, pre‐treatment patient‐specific VMAT PD‐QA was performed. The QA pass rate was 99.3% for 2%/2 mm gamma criteria as shown in Figure 2. The BOT recorded during PD‐QA was clocked in at 3.26 min. Moreover, independent Monte Carlo 2^nd^ physics check results agreed within 1% when compared to Acuros‐based Eclipse planned dose distribution on the Halcyon RDS.

**FIGURE 3:**
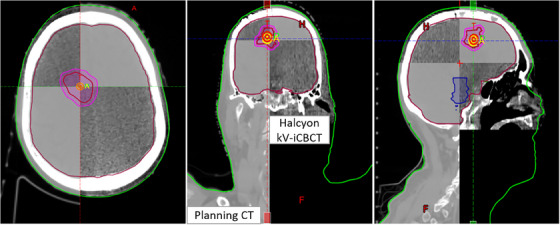
Axial, coronal, and sagittal views of the Halcyon kV‐iCBCT images (see split window in all 3 views) co‐registered with planning CT images used for image guided‐SRT on Halcyon. On top of anatomical landmarks, the GTV (red) and PTV (pink) are shown. The Halcyon kV‐iCBCT images were acquired in the treatment position, 3D rigid‐registration was performed via auto‐matching of online kV‐iCBCT with the planning CT followed by manual refinement of the registration. Planning isocenter (P) and CBCT acquisition isocenter (A) are shown. The setup errors in the first treatment were <2 mm in each direction.

On the treatment day, the patient was initially positioned using external marks on the long head and shoulder mask and in‐room virtual blue lasers, followed by the “one‐step patient setup and verification” and a 15‐s pre‐treatment kV‐iCBCT scan on the Halcyon. An in‐house IGRT/SBRT clinical protocol was applied to co‐register the kV‐iCBCT images with the planning images shown in Figure 3. Image registration was performed automatically based on bony anatomy followed by additional corrections and reviewed by the attending physician and physicist. The patient's setup was corrected translationally with the Halcyon's 3DoF couch and was within the limits of departmental SRT protocols (treatment translational shifts within ±3.0 mm in each direction). The net beam‐on time was 3.26 min with an overall patient's door‐to‐door treatment time of less than 7 min.

## 4 DISCUSSION

In this technical report, we have evaluated the plan quality, treatment delivery efficiency, and treatment accuracy of SRT plans for intracranial resected tumor beds on the Halcyon RDS by benchmarking against the clinical TrueBeam HyperArc plans. All treatment plans followed our clinical SRT protocol, which were based on TG‐101 recommendations, Alliance brain 071801 trial, and HyTEC brain SRT papers recommendations. Our dosimetric study has found that for select patients, the Halcyon is capable of delivering high quality, intracranial SRT treatments that are similar to clinical HyperArc plans in terms of plan quality and delivery accuracy. Although the Halcyon did demonstrate inferior GI and D_2cm_ due to its coplanar geometry compared to the highly non‐coplanar HyperArc geometry, it still met all the clinical protocols requirements for brain SRT treatments. Furthermore, due to its coplanar geometry, our results suggest that the Halcyon provides a statistically significant decrease in maximum dose to the optic pathways that was higher when utilizing the HyperArc non‐coplanar geometry. This is a result of the Hyperarc's non‐coplanar geometry often having the beam enter or exit through the optic pathways. Even though the Halcyon demonstrated a relatively longer BOT, we calculated an average treatment time (patient's door‐to‐door time) of 7.61 min, an absolute decrease of around 2.5 min compared to patients treated with HyperArc on the TrueBeam LINAC. These treatment times on the Halcyon are based on the recorded timings of the patients we have treated. This reduction in treatment time is credited to the Halcyon's automated one‐step patient setup, 4 times faster gantry rotation speed, two times faster MLC speed, as well having no need to apply couch rotations that the HyperArc plans needed on the TrueBeam LINAC.

In the past few years, at least a few studies have reported the possibility of utilizing the Halcyon RDS in providing high quality intracranial SRT treatments as we mentioned earlier.^23^, ^24^ Similar to our work, Knutson et al.^23^ developed and tested a clinical workflow to plan and deliver brain SRT treatments on the Halcyon and show that the Halcyon was able to provide similar plan quality to that of the clinical Varian EDGE LINAC's plans using a 30 Gy in five fractions regimen. Our study also dosimetrically studied the feasibility of using the Halcyon for intracranial SRT, in where they had more cases used for dosimetric analysis. However, where our study differs is that we have included some typical outliers for patient's plan evaluation on Halcyon with OAR near the target or in the same plane and a prospective case report is presented that will be useful for other Halcyon users to reference during validation and clinical implementation of Halcyon for brain SRT into their clinic. Additionally, Li et al.^24^ performed a retrospective study of the Halcyon and found out that it can provide similar plan quality compared to the TrueBeam for treating multiple brain lesions (6 to 10) with a single‐isocenter/multi‐target plan. However, these aforementioned reports only retrospectively demonstrated acceptable plan quality, none of these plans were used in treating actual brain SRT patients on Halcyon in the clinical setting. Moreover, treating 6−10 brain lesions via single‐isocenter VMAT SRT plan on Halcyon will be challenging due to the spatial patient setup uncertainty. Our benchmarking study on Halcyon and for prospectively treating select brain SRT patients with one large tumor bed complements these previous dosimetry planning studies.

This study shows promise for the Halcyon's utility in SRT treatments for intracranial tumor beds, but there are some limitations. One major limitation of the current Halcyon is its limited 3DoF couch correction compared to the SBRT‐dedicated TrueBeam 6DoF PerfectPitch couch corrections. As of now, the dosimetric impact of the 3DoF couch compared to the 6DoF couch for large targets are not fully characterized and is an avenue for further investigation. Another limitation is that our study has only included patients treated with a regimen of 30–40 Gy in five fractions. Based on tumor size and location, more investigations are needed to understand which fractionation schemes are possible on the Halcyon RDS for these treatments including the most commonly used dosing scheme of 27 Gy in three fractions brain SRT in our clinic; or is single dose of brain SRS treatment possible on current Halcyon? Also, in this study we have not further investigated the total optimal number of VMAT arcs to be used for generating the optimal brain SRT plan on Halcyon while keeping the similar BOT, we are planning to further explore in the future. Moreover, our Halcyon does not yet have a full package of intrafraction motion monitoring system clinically implemented. Our final limitation to note is that the patients on Halcyon will be restricted by tumor size. That is due to Halcyon's lack of output factors for less than 1 × 1 cm^2^ MLC field sizes, currently, it is not possible to calculate the SRT plans accurately for these targets smaller than 1 cm in diameter on Halcyon.

Overall, in this benchmarking technical report, we have demonstrated the feasibility of the Halcyon RDS in providing SRT treatments for intracranial tumor beds. This study showed that the Halcyon can provide high‐quality brain SRT treatments similar to that of the clinical TrueBeam plans generated via the highly non‐coplanar HyperArc geometry. As mentioned above, based on our promising clinical validation and testing results, we have started treating selected brain SRT patient on the Halcyon. Clinical follow‐up on the prospective patients’ treatments is underway. Our ongoing research includes computing and analyzing the dosimetric impacts of rotational patient set up errors and monitoring intra‐ and interfraction motion errors for large, intracranial tumor beds SRT on Halcyon RDS.

## 5 CONCLUSION

Our validation study demonstrated the potential utility of the Halcyon RDS in providing high quality brain SRT plans for large tumor beds including large resection cavities that were similar to the TrueBeam clinical HyperArc plans. These dosimetric and treatment delivery results suggests that the Halcyon could provide safe, effective, and accurate SRT treatments to intracranial patient with an average door‐to‐door time of 7.61 min. We have started treating brain SRT patients on the Halcyon in our clinic and we encourage other Halcyon users to benchmark and implement SRT treatments on their Halcyon, especially for Halcyon‐only clinics. The patient selection criteria we utilize is that the tumor bed should be greater than 1 cm in diameter, and nearby critical organs should be at least 2 cm away from the target. That will allow for an increase access of therapeutic SRT treatments for remote and underserved patient cohorts, expanding the outreach of an exceptional quality of brain SRT treatments including developing countries.

## AUTHOR CONTRIBUTIONS

Damodar Pokhrel conceived the project and generated the treatment plans. Joshua Misa and Shane McCarthy collected and analyzed the data. William St. Clair provided clinical expertise and supervision of this project. Joshua Misa and Damodar Pokhrel drafted the preliminary manuscript and all co‐authors revised and approved the final manuscript for submission.

## CONFLICT OF INTEREST STATEMENT

The authors declare no conflicts of interest.
